# Prevalence and determinants of cardiovascular disease risk factors using the WHO STEPS approach in Cochabamba, Bolivia

**DOI:** 10.1186/s12889-019-7064-y

**Published:** 2019-06-20

**Authors:** Yercin Mamani-Ortiz, Miguel San Sebastián, Ada X. Armaza, Jenny M. Luizaga, Daniel E. Illanes, Marcia Ferrel, Paola A. Mosquera

**Affiliations:** 1 Biomedical and Social Research Institute, Faculty of Medicine, San Simon University, Aniceto Arce Avenue, 371 Cochabamba, Bolivia; 2 Department of Epidemiology and Global Health, Umeå University, Umeå, Sweden; 3 Departmental Health Service, Cochabamba, Bolivia

**Keywords:** WHO STEPS approach, Cardiovascular risk factors, Obesity, Hypertension, Tobacco, Alcohol, Bolivia

## Abstract

**Background:**

Cardiovascular diseases (CVDs) are considered the number one cause of death worldwide, especially in low- and middle-income countries, Bolivia included. Lack of reliable estimates of risk factor distribution can lead to delay in implementation of evidence-based interventions. However, little is known about the prevalence of risk factors in the country. The aim of this study was to assess the prevalence of preventable risk factors associated with CVDs and to identify the demographic and socioeconomic factors associated with them in Cochabamba, Bolivia.

**Methods:**

A cross-sectional community-based study was conducted among youth and adults (*N* = 10,704) with permanent residence in Cochabamba, selected through a multistage sampling technique, from July 2015 to November 2016. An adapted version of the WHO STEPS survey was used to collect information. The prevalence of relevant behavioural risk factors and anthropometric measures were obtained. The socio-demographic variables included were age, ethnicity, level of education, occupation, place of residence, and marital status. Proportions with 95% confidence intervals were first calculated, and prevalence ratios were estimated for each CVD risk factor, both with crude and adjusted models.

**Results:**

More than half (57.38%) were women, and the mean age was 37.89 ± 18 years. The prevalence of behavioural risk factors were: current smoking, 11.6%; current alcohol consumption, 42.76%; low consumption of fruits and vegetables, 76.73%; and low level of physical activity, 64.77%. The prevalence of overweight was 35.84%; obesity, 20.49%; waist risk or abdominal obesity, 54.13%; and raised blood pressure, 17.5%. Indigenous populations and those living in the Andean region showed in general a lower prevalence of most of the risk factors evaluated.

**Conclusion:**

We provide the first CVD risk factor profile of people living in Cochabamba, Bolivia, using a standardized methodology. Overall, findings suggest that the prevalence of CVD risk factors in Cochabamba is high. This result highlights the need for interventions to improve early diagnosis, monitoring, management, and especially prevention of these risk factors.

## Background

The worldwide epidemic of non-communicable diseases (NCD) is well known, with cardiovascular diseases (CVDs) the most frequent and the number one cause of death in the world [1, 2]. The magnitude of these diseases is higher in low- and middle-income countries [3], representing 13.6% of the total estimated disability adjusted life years (DALYs) between 2000 and 2012, compared with 2.8% in high-income countries (HICs) [4].

Evidence worldwide suggests that a large proportion of CVD cases can be prevented if risk factors are controlled [3, 5]. The prevalence of CVD risk factors in Latin America (LA) is considerably high, with 57.1% of men and 58.3% of women being overweight or obese, 7.5% heavy drinkers, 23.8% of men and 18.0% of women having high blood pressure, 15.8% reporting to be current smokers, and 31.2% of adults characterized by insufficient physical activity [6]. These risk factors are modifiable, and thus their continuing surveillance is fundamental for CVD control [5, 7].

The Pan American Health Organization (PAHO) has reported that NCDs are responsible for 59% of the overall mortality in Bolivia, and CVDs alone for 24% of the total mortality [8]. However, these figures are only estimates, and more accurate information is needed to support decision making. Indeed, the lack of accurate information about CVD prevalence and associated risk factors is one of the major difficulties for the implementation of preventive local health programs in the country [7, 9]. The only available data comes from the National Health Information System (NHIS), which has a registration bias, since it only captures patients who come to the public health system, leaving aside users of private health care, or people who have not accessed the health care system [9]. Since the NHIS prioritizes infectious diseases and maternal and child health, only information regarding diabetes, hypertension, obesity, cancer (any type), and rheumatoid arthritis is collected [9]. Moreover, the planning units of the Departmental Health Services and municipal governments do not have estimates of the magnitude of the problem locally, and therefore no prioritized interventions based on their own population characteristics can be properly implemented [9–11]. The existing studies in Bolivia have reported a high prevalence of obesity (60.7%) [12], high blood pressure (36%) [13], alcohol consumption (85%) [14], and sedentarism (67.2%) [15]. Nevertheless, these studies were focused on a limited number of risk factors, several were hospital-based, and none of them used the World Health Organization (WHO) STEPS methodology (Surveillance of Noncommunicable Diseases), so that they lacked a comprehensive picture of current cardiovascular and behavioural risk factors at the population level.

This study aimed to assess the prevalence of preventable risk factors associated with CVDs and to identify the demographic and socioeconomic factors associated with them by using the STEPS approach in Cochabamba, Bolivia. The development of a risk factor profile for CVDs will provide key information required for planning prevention and control activities as well as to help predict the future burden of disease.

## Methods

### Study setting and participants

Cochabamba is one of the nine departments of Bolivia, located in the centre of the Andes mountain range. In 2012, demographic data indicated that 1.8 million people lived in this department, representing 17.5% of the national population; approximately 35–40% of them lived in rural areas [16].

Cochabamba is divided into five different socio-demographic regions: the Central Valley, which includes the capital city and other municipalities of the metropolitan area; the High Valley region, which is a semi-humid agricultural area; the Andean region, located in the Andes mountain range above 3500 m; the Southern Cone region, which comprises the areas of dry or semi-arid valleys; and the tropics, which include the Amazon rainforest. Cochabamba’s geography is varied, and lifestyles of people have been modified over time, particularly by internal migration flows.

### Study design, population and sampling methodology

A cross-sectional community-based study was conducted among youth and adults (18 years and older) with permanent residence in urban and rural areas of Cochabamba, from July 2015 to November 2016.

The sample size (*N* = 10,609) was calculated based on previous estimates of the prevalence of overweight and obesity in the department (around 30%) using a level of confidence of 5%, a margin of error of 0.05, and a design effect of 1.05 as recommended by the STEPS manual [7]. Assuming a response rate of 85%, the target sample size was raised to 12,779.

A proportionate allocation as per census distribution in all municipalities was adopted. A list of 47 municipalities, 437 primary health care service areas (PHCSAs), and 968 communities comprised our sampling frame. In a first stage, the intervention area of the primary health care centres was divided into sub-areas with a similar population size proportional to the sample size of each PHCSA. In a second stage, a population sampling unit (PSU) (either village, district, or neighbourhood, following the official classification for Bolivia) was randomly selected from each sub-area. In the final stage, households were randomly selected within each PSU using a systematic random sampling procedure. The ultimate sampling units were the households where one individual 18 years or older was selected using the Kish method [17]. One inclusion criterion considered for a person to be selected was to have been living in the community for at least the last six months prior to the survey. This criterion was applied due to the great social and geographical mobility that characterizes the population of rural areas of the country. Critically ill patients, pregnant women, patients with ascites, and those who did not consent were excluded. The sample of eligible subjects consisted of 12,527 persons, of whom 85.45% participated in the study. The final sample used for analysis comprised 10,704 individuals.

### Data collection and measurements

The data collection procedure was based on the Pan American version (V2.0) of the WHO STEPS approach [7] adapted to the Bolivian context. The STEPS approach follows three stages: a) Step 1 uses a questionnaire to collect demographic and lifestyle data; b) Step 2 involves measurements of height, weight, blood pressure, and waist circumference; and c) Step 3 uses biochemical assessments. Some items in the Step 1 questionnaire were reformulated to use Bolivian expressions, and also new contextual questions were added (e.g., questions about type of alcohol and tobacco, type of fruits and vegetables, etc.), all in accordance with the WHO STEPS manual. The adapted version was pretested with a group of military personnel (*N* = 204) to identify practical problems, and modifications were conducted when necessary [14].

The STEPS tools were applied by a group of health personnel from the PHCAS through direct interviews. All interviewers underwent training for two days, which covered the three stages of STEPS, including classroom interactive sessions and skill development for interviews and field visits. Pilot testing for applying the instrument for Steps 1 and 2 (pretested) was conducted with the same staff, who helped to develop an application guide.

In Step 1, a structured questionnaire was used for face-to-face interviews. Participants were asked about demographic information including age (categorized into four groups according to the Global Burden of Disease-GBD: 18–29, 30–44, 45–59, and ≥ 60 years); gender (defined as male or female); marital status (never married, currently married, or cohabitation/widowed/separated); education level (categorized into four groups: no formal schooling, primary school, secondary school, and higher education); ethnicity (categorized into three groups: indigenous—Quechua and Aymara, mestizos, and white/black as others); occupation (classified into five groups: self-employed, employed, housewife or homemaker, retired, and unemployed); and place of residence (according to the five socio-demographic regions: Andean, Southern cone, Central Valley, Tropics, and High Valley). All categorizations were based on the STEPS manual [7].

Information about risk factors was also collected, including fruit and vegetable intake (less than five servings or approximately 200 g of fruits and vegetables per day were considered as the ‘at risk’ group); tobacco use (having smoked in the past 30 days), alcohol consumption (amount, frequency, and patterns of drinking in the past month); and physical activity in their daily lives. Physical activity was measured using the Global Physical Activity Questionnaire format (part of the STEPS tool), and information was gathered about four different aspects: physical activity at the workplace, during recreation time, while travelling, and during resting time. Based on the Metabolic Equivalent of Task (MET), a value less than 600 MET-minutes per week was classified as low physical activity, and values higher than 600 MET-minutes per week were classified as appropriate [7, 18].

In Step 2, measurements were done using calibrated and standardized instruments. Physical measurements included weight (in bare feet without heavy clothing, in consideration of cultural principles) and height (in bare feet and without headwear); with this data, the Body Mass Index (BMI) was calculated, and the participants were classified as overweight (BMI between 25 and 29.9 kg/m2) and obese (BMI ≥ 30 kg/m2). For older persons, the BMI parameters of the Spanish Society of Geriatrics and Gerontology and the Spanish Society of Parenteral and Enteral Nutrition were used [19]. Waist circumference was measured at the narrowest point between the lower costal border and the iliac crest using a constant-tension tape (abdominal obesity being defined as a waist circumference of > 90 cm in men and > 80 cm in women). Blood pressure was measured at the midpoint of both arms after participants had rested for at least five minutes. Two blood pressure readings were obtained from all participants. A third reading was taken if there was a difference of more than 25 mmHg for systolic blood pressure or 15 mmHg for diastolic blood pressure between the first two readings. The mean of all measures was used, based on the recommendations of the WHO research protocol. Raised blood pressure was defined as a systolic blood pressure of ≥130 mm/Hg, or a diastolic blood pressure of ≥85 mm/Hg or the self-reported use of anti-hypertensive medications, based on the WHO and American College of Cardiology guidelines [5, 7]. All instruments were standardized before the examination, and the scales were zero calibrated routinely during the study period. Step 3 was performed only in the capital city, and results have not been included in this manuscript.

Questionnaires with missing or conflicting information were sent back to be rechecked and completed, and the research team conducted a random verification of the collected data through telephone calls by selecting one survey for every 100 participants.

### Statistical methods

Data were entered into MS Excel and then transferred into Stata/MP version 14.0 (StataCorp) for data cleaning and analysis. Prevalence of cardiovascular risk factors by age, gender, marital status, education, ethnicity, occupation, and place of residence is presented in percentages with 95% confidence intervals (CIs). Crude and adjusted prevalence ratios were estimated for each CVD risk factor, through generalized linear models with a binomial distribution and a log link. For the adjusted model, we include all covariates simultaneously in the model.

## Results

Table 1 describes the socio-demographic characteristics of the participants by gender. More than half (57.38%) were women, and the mean age was 37.89 ± 18 years (women = 36.88 ± 17.58 and men = 39.24 ± 18.62). The majority of the participants were living in the Central (37.89%) and the High (31.07%) Valley regions. Most of the study population (91.49%) received formal education in different grades, and 64.33% self-identified as indigenous. More than half (60.22%) were married or cohabitating, and 50.03% were working (self-employee and government or non-government employee).

**Table 1:** Socio-demographic information on participants in study of cardiovascular disease risk factors, Cochabamba, Bolivia, 2015–2016

Socio-demographic variables	Female (*N* = 6143 - 57,39%)		Male (*N* = 4561 - 42,61%)		Both Genders (*N* = 10,704)	
	n	%	n	%	n	%
Age group						
18–29	2759	44.91	1843	40.41	4602	42.99
30–44	1597	26.00	1102	24.16	2699	25.21
45–59	864	14.06	758	16.62	1622	15.15
≥ 60	923	15.03	858	18.81	1781	16.65
Residence						
Andean	575	9.36	462	10.13	1037	9.69
Southern cone	375	6.10	268	5.88	643	6.01
Central Valley	2232	36.33	1824	39.99	4056	37.89
Tropics	922	15.01	720	15.79	1642	15.34
High Valley	2039	33.19	1287	28.22	3326	31.07
Education						
No formal schooling	646	10.52	265	5.81	911	8.51
Primary school	2691	43.81	1811	39.71	4502	42.06
Secondary school	2119	34.49	1849	40.54	3968	37.07
Higher education	687	11.18	636	13.94	1323	12.36
Ethnicity						
Indigenous	4080	66.42	2806	61.52	6886	64.33
Mestizo	2015	32.80	1694	37.14	3709	34.65
Other	48	0.78	61	1.34	109	1.02
Marital Status						
Never married	1782	29.01	1581	34.66	3363	31.42
Currently married or cohabitating	3794	61.76	2652	58.15	6446	60.22
Widowed or separated	567	9.23	328	7.19	895	8.36
Occupation/labour market position/status						
Student	964	15.81	743	16.45	1707	16.08
Self-employed	1621	26.59	2742	60.7	4363	41.11
Employed	557	9.14	708	15.67	1265	11.92
Housewife or homemaker	2776	45.53	34	0.75	2810	26.47
Retired	81	1.33	182	4.03	263	2.48
Unemployed	98	1.61	108	2.39	206	1.94

Table 2 presents the prevalence of behavioural risk factors by socio-demographic factors. Smoking prevalence overall was 11.06%, being lower in women (3.25%) than in men (21.57%). Men (21.7%), people of the tropic region (15.83%), the most educated (14.13%) and those currently working (self-employed = 17.19% and employee = 15.88%) had the highest smoking prevalence.

**Table 2:** Prevalence of cardiovascular disease risk factors stratified by socio-demographic variables, Cochabamba, Bolivia, 2015–2016

STEP 1: Behavioural Risk Factors (%, 95 CI)				
Socio-demographic variables	Current daily smoker	Current alcohol consumption	Low fruit and vegetable consumption	Low level of physical activity
Gender				
Female	3.25 (2.81–3.69)	33.89 (32.70–35.07)	76.29 (75.23–77.36)	72.78 (71.66–73.89)
Male	21.57 (20.38–22.76)	54.72 (53.27–56.16)	77.32 (76.11–78.54)	53.97 (52.53–55.42)
Age group				
18–29	10.53 (9.65–11.42)	37.87 (36.47–39.27)	75.18 (73.93–76.43)	67.90 (66.55–69.25)
30–44	12.22 (10.99–13.46)	50.64 (48.76–52.53)	76.32 (74.72–77.92)	58.83 (56.97–60.69)
45–59	12.39 (10.78–13.99)	50.73 (48.30–53.17)	76.75 (74.70–78.81)	58.69 (56.29–61.09)
≥ 60	9.43 (8.07–10.79)	36.21 (33.98–38.44)	81.35 (79.54–83.16)	71.19 (69.09–73.29)
Residence				
Andean	11.37 (9.44–13.31)	35.39 (32.47–38.30)	80.32 (77.90–82.74)	59.98 (56.99–62.96)
Southern cone	12.13 (9.60–14.65)	55.05 (51.20–58.90)	83.04 (80.14–85.95)	54.74 (50.89–58.59)
Central Valley	11.80 (10.81–12.80)	54.51 (52.97–56.97)	72.14 (70.76–73.52)	66.98 (65.53–68.43)
Tropics	15.83 (14.06–17.60)	54.56 (52.15–56.97)	76.85 (74.81–78.89)	51.52 (49.10–53.94)
High Valley	7.48 (6.59–8.38)	59.98 (58.31–61.64)	79.94 (78.58–81.30)	72.03 (70.51–73.56)
Ethnicity				
Indigenous	10.57 (9.84–11.29)	41.89 (40.73–43.06)	79.01 (78.05–79.97)	62.08 (60.93–63.22)
Mestizo	11.97 (10.92–13.01)	44.32 (42.72–45.92)	72.93 (71.50–74.36)	69.69 (68.21–71.17)
Other	11.00 (5.10–16.91)	44.95 (35.57–54.33)	62.38 (53.24–71.52)	66.97 (58.10–75.84)
Education				
No formal schooling	6.69 (5.07–8.32)	30.51 (27.52–33.50)	83.86 (81.47–86.25)	65.97 (62.89–69.05)
Primary school	10.28 (9.39–11.17)	41.51 (40.07–42.95)	78.76 (77.57–79.95)	61.32 (59.90–62.75)
Secondary school	11.92 (10.91–12.92)	42.08 (40.55–43.62)	75.32 (73.98–76.66)	66.53 (65.06–68.00)
Higher education	14.13 (12.25–16.01)	57.52 (54.85–60.18)	30.83 (28.34–33.32)	70.37 (67.90–72.83)
Marital status				
Never married	11.86 (10.77–12.95)	37.82 (36.18–39.46)	76.00 (74.55–77.44)	68.74 (67.18–70.31)
Currently married or cohabitating	10.81 (10.05–11.57)	45.84 (44.62–47.05)	76.57 (75.54–77.60)	61.85 (60.66–63.03)
Widowed or separated	9.83 (7.88–11.78)	39.21 (36.01–42.41)	80.67 (78.08–83.25)	70.83 (67.85–73.81)
Occupation				
Student	6.56 (5.38–7.73)	25.71 (23.64–27.79)	74.45 (72.38–76.52)	78.44 (76.49–80.39)
Self-employed	17.19 (16.07–18.30)	51.89 (50.40–53.37)	77.72 (76.48–78.95)	49.00 (47.51–50.48)
Employed	15.88 (13.87–17.90)	58.81 (56.10–61.52)	72.80 (70.35–75-25)	64.18 (61.54–66.83)
Housewifee or homemaker	2.24 (1.69–2.78)	32.98 (31.25–34.72)	78.07 (76.54–79.60)	77.04 (75.49–78.60)
Retired	10.64 (6.91–14.38)	39.16 (33.25–45.07)	76.04 (70.87–81.21)	87.83 (83.87–91.79)
Unemployed	8.73 (4.87–12.60)	31.55 (25.19–37.91)	77.18 (71.43–82.92)	86.89 (82.27–91.51)
Overall	11.06 (10.46–11.65)	42.76 (41.83–43.70)	76.73 (75.93–77.53)	64.77 (63.86–65.67)
STEP 2: Physical Measurements (%, 95 CI)				
Socio-demographic variables	Overweight	Obesity	Abdominal obesity	Raised blood pressure
Gender				
Female	35.17 (33.91–36.43)	23.97 (22.84–25.09)	64.12 (62.86–65.39)	14.32 (13.40–15.25)
Male	36.75 (35.28–38.22)	15.83 (14.72–16.94)	40.21 (38.71–41.17)	21.22 (19.98–22.47)
Age group				
18–29	30.66 (29.25–32.06)	10.38 (9.45–11.31)	39.10 (37.61–40.59)	8.85 (7.99–9.72)
30–44	41.50 (39.55–43.46)	28.85 (27.06–30.65)	65.67 (63.78–67.55)	16.90 (15.41–18.39)
45–59	39.54 (37.07–42.05)	32.48 (27.06–30.65)	69.54 (67.18–71.90)	26.96 (24.69–29.24)
≥ 60	37.20 (34.85–39.54)	22.83 (20.79–24.87	59.72 (57.34–62.11)	30.50 (28.27–32.74)
Residence				
Andean	38.83 (35.66–42.00)	7.37 (5.67–9.07)	41.80 (38.59–45.01)	10.01 (8.05–11.96)
Southern cone	40.91 (36.94–44.89)	16.46 (13.47–19.46)	52.63 (48.59–56.66)	26.99 (23.40–30.58)
Central Valley	35.36 (33.81–36.90)	22.04 (20.70–23.38)	54.10 (52.48–55.71)	18.06 (16.81–19.30)
Tropics	33.14 (30.68–35.60)	33.14 (30.68–35.60)	61.69 (59.15–64.23)	17.63 (15.64–19.63)
High Valley	35.81 (34.12–37.50)	20.13 (18.72–21.54)	53.89 (52.13–55.65)	16.48 (15.17–17.79)
Ethnicity				
Indigenous	35.63 (34.44–36.82)	19.80 (18.81–20.79)	53.76 (52.53–55.00)	17.17 (16.24–18.11)
Mestizo	36.37 (34.74–38.01)	21.74 (20.34–23.15)	54.13 (52.44–55.83)	17.33 (16.05–18.62)
Other	31.31 (22.13–40.49)	21.21 (13.11–23.90)	53.53 (43.65–63.41)	21.21 (13.11–29.30)
Education				
No formal schooling	34.84 (31.57–38.10)	18.94 (16.26–21.63)	60.14 (56.78–63.50)	23.59 (20.68–26.50)
Primary school	36.17 (34.70–37.64)	23.40 (22.11–24.70)	57.09 (55.58–58.61)	18.03 (16.85–19.21)
Secondary school	34.33 (32.77–35.88)	17.23 (15.99–18.47)	47.74 (46.11–49.38)	14.32 (13.17–15.47)
Higher education	40.03 (37.22–42.83)	21.29 (18.95–23.63)	57.06 (54.22–59.88)	19.21 (16.96–21.47)
Marital Status				
Never married	28.32 (26.71–29.93)	10.22 (9.14–11.31)	34.99 (33.29–36.70)	11.52 (10.38–12.67)
Currently married cohabitating	39.48 (38.23–70.73)	24.82 (23.71–25.93)	61.95 (60.71–63.19)	18.63 (17.63–19.63)
Widowed or separated	37.39 (34.02–40.75)	27.22 (24.13–30.31)	65.74 (62.44–69.04)	28.98 (25.83–32.13)
Occupation				
Student	22.68 (20.58–24.78)	5.28 (4.16–6.39)	26.49 (24.28–28.70)	7.16 (5.87–8.45)
Self-employed	38.65 (37.13–40.17)	22.07 (20.77–23.37)	54.25 (52.70–55.81)	20.34 (19.08–21.60)
Employed	39.38 (36.54–42.22)	20.26 (17.92–22.59)	57.40 (54.53–60.27)	18.22 (15.98–20.47)
Housewife or homemaker	37.33 (35.46–39.21)	27.71 (25.97–29.44)	68.00 (66.20–69.81)	15.99 (14.57–17.41)
Retired	43.80 (37.53–50.06)	18.59 (13.68–23.50)	57.43 (51.19–63.68)	38.84 (32.68–44.99)
Unemployed	34.25 (27.32–41.18)	19.33 (13.56–25.10)	56.35 (49.10–63.59)	19.33 (13.56–25.10)
Overall	35.84 (34.89–36.80)	20.49 (19.68–21,29)	54.13 (53.17–55.08)	17.15 (16.44–17.87)

The overall prevalence of current alcohol consumption was 42.76%. Men (54.72%), people aged 30–44 and 45–59 years old (> than 50%), non-indigenous groups (around 44%), people with higher education (57.52%), currently married or cohabitating (45.84%), and employees (58.81%) had the highest prevalence, while women (33.89%), students (25.71%), and people of the Andean region (35.39%) presented the lowest prevalence (Table 2).

Low levels of fruit and vegetable intake were present in 76.73% of people. This low-intake prevalence was high among all socio-demographic groups (above 72%), except among those with higher education (30.83%). A similar pattern was observed regarding the low levels of physical activity, with an overall prevalence of 64.77%. The prevalence was above 50% in all socio-demographic groups and very high in the retired and unemployed population (87.83 and 86.89%, respectively) (Table 2).

Overweight and obesity were observed in 35.84 and 20.49% of participants, respectively. Prevalence of overweight was similar among women and men. Singles and students had the lowest prevalence (28.32 and 22.68%, respectively), while those aged 30–44 years (41.50%), living in the Southern Cone region (40.91%), with higher education (40.03%), and retired (43.80%) had the highest prevalence. Unlike overweight, obesity was more prevalent in women (23.97%) and the 45–59 years age group (32.48%) as well as among those living in the tropic region (33.14%). The lowest prevalence was found among those who lived in the Andean region (7.37%) or who belonged to the student group (5.28%) (Table 2).

Central obesity (abdominal obesity) was present in 54.13% of the participants, being higher among women (64.12%) than men (40.21%). It was also higher among people aged 30–44 and 45–59 years (65.67% and 69.54, respectively). The Andean region presented a low prevalence (41.80%) compared to other regions (above 52%); similarly, singles and students (34.99 and 26.49%, respectively) presented a low prevalence compared to the other subgroups. The prevalence was similar among the ethnic groups (around 54%) and higher among those with no formal schooling (60.14%), widowed or separated (65.74%), and housewives/homemakers (68%). (Table 2).

The overall prevalence of raised blood pressure was 17.15%, being higher among men (21.22%), people aged over 60 years (30.50%), and housewives/homemakers (38.84%). The prevalence was lower in the 30–44 years age group (8.85%), those who lived in the Andean region (10.1%), and those who belonged to the student group (7.16%). (Table 2).

Table 3 shows the probability of presenting the risk factors in the different socio-demographic groups. The adjusted prevalence ratios are presented in the Table 3. After adjustment, men were found to have significantly higher risk of smoking (PR: 6.62, 95% CI: 5.71–7.67), alcohol consumption (PR: 1.61, 95% CI: 1.54–1.68), and raised blood pressure (PR: 1.48, 95% CI: 1.36–1.61), but lower risk of being overweight or obese (PR: 0.88, 95% CI: 0.85–0.91), having abdominal obesity (PR: 0.62, 95% CI: 0.60–0.65), and having low levels of physical activity (PR: 0.74, 95% CI: 0.0.71–0.76) than women (Table 3).

**Table 3:** Prevalence Ratio (PR) of cardiovascular disease risk factors stratified by socio-demographic variables, Cochabamba, Bolivia, 2015–2016

	STEP 1: Behavioural Risk Factors (PR, 95% CI)				SETEP 2: Physical Measurements (PR, 95% CI)		
Socio-demographic variables	Current daily smoker	Current alcohol consumption	Low fruit and vegetable consumption	Low level of physical activity	Overweight and obesity	Abdominal obesity	Raised blood pressure
Gender							
Female	Reference category						
Male	6.62 (5.71–7.67) *	1.61 (1.54–1.68) *	1.01 (0.99–1.03)	0.74 (0.71–0.76) *	0.88 (0.85–0.91) *	0.62 (0.60–0.65) *	1.48 (1.36–1.61) *
Age group							
18–29	Reference category						
30–44	1.16 (1.01–1.32) *	1.33 (1.26–1.40) *	1.01 (0.98–1.04)	0.86 (0.83–0.89) *	1.71 (1.63–1.78) *	1.66 (1.58–1.73) *	1.84 (1.63–2.09) *
45–59	1.17 (1.00–1.37) *	1.33 (1.26–1.42) *	1.02 (0.98–1.05)	0.86 (0.82–0.90) *	1.74 (1.66–1.83) *	1.75 (1.67–1.84) *	2.98 (2.64–3.38) *
≥ 60	0.89 (0.75–1.05)	0.95 (0.88–1.02)	1.08 (1.05–1.11) *	1.04 (1.01–1.08) *	1.44 (1.37–1.52) *	1.50 (1.42–1.58) *	3.43 (3.05–3.85) *
Residence							
Andean	Reference category						
Southern cone	1.06 (0.81–1.39)	1.26 (1.12–1.42) *	1.03 (0.98–1.08)	0.91 (0.83–0.99) *	1.26 (1.14–1.39) *	1.29 (1.16–1.43) *	2.83 (2.25–3.56) *
Central Valley	1.03 (0.85–1.25)	1.28 (1.17–1.40) *	0.89 (0.86–0.93) *	1.11 (1.05–1.17) *	1.25 (1.16–1.35) *	1.31 (1.21–1.42) *	1.87 (1.53–2.28) *
Tropics	1.39 (1.13–1.70) *	1.28 (1.16–1.41) *	0.95 (0.91–0.99) *	0.85 (0.80–0.91) *	1.32 (1.22–1.44) *	1.49 (1.37–1.62) *	1.86 (1.50–2.31) *
High Valley	0.65 (0.53–0.80)	1.13 (1.03–1.23) *	0.99 (0.96–1.03) *	1.20 (1.13–1.26) *	1.23 (1.14–1.33) *	1.31 (1.21–1.42) *	1.74 (1.42–2.14) *
Ethnicity							
Mestizo and other	Reference category						
Indigenous	0.88 (0.79–0.98) *	0.94 (0.90–0.98) *	1.08 (1.06–1.11) *	0.89 (0.86–0.91) *	0.96 (0.92–0.99) *	0.98 (0.95–1.02)	0.98 (0.89–1.06)
Education							
No formal schooling	0.47 (0.35–0.62) *	0.53 (0.47–0.59) *	1.21 (1.15–1.26) *	0.93 (0.88–0.99) *	0.87 (0.88–0.94) *	1.06 (0.99–1.14)	1.24 (1.05–1.46) *
Primary school	0.72 (0.62–0.85) *	0.72 (0.68–0.76) *	1.13 (1.09–1.18) *	0.87 (0.83–0.90) *	0.98 (0.93–1.03)	1.00 (0.95–1.06)	0.97 (0.85–1.10)
Secondary school	0.84 (0.72–0.98) *	0.73 (0.68–0.77) *	1.08 (1.04–1.13) *	0.94 (0.90–0.98) *	0.84 (0.80–0.89) *	0.84 (0.79–0.89) *	0.74 (0.65–0.85) *
Higher education	Reference category						
Marital Status							
Never married	Reference category						
Currently married or cohabitating	0.91 (0.81–1.02)	1.21 (1.15–1.27) *	1.00 (0.98–1.03)	0.89 (0.87–0.92) *	1.67 (1.59–1.75) *	1.75 (1.66–1.84) *	1.61 (1.45–1.80) *
Widowed or separated	0.82 (0.66–1.03)	1.03 (0.94–1.13)	1.06 (1.02–1.10) *	1.03 (0.98–1.08)	1.67 (1.56–1.79) *	1.84 (1.72–1.96) *	2.48 (2.15–2.85) *
Occupation/labour market position/status							
Student	Reference category						
Self-employed	2.61 (2.16–3.16) *	2.01 (1.85–2.19) *	1.04 (1.01–1.07) *	0.62 (0.60–0.64) *	2.16 (1.98–2.35) *	2.04 (1.87–2.22) *	2.88 (2.40–3.46) *
Employed	2.42 (1.94–3.01) *	2.28 (2.08–2.50) *	0.97 (0.93–1.02)	0.81 (0.77–0.85) *	2.11 (1.92–2.32) *	2.15 (1.96–2.36) *	2.57 (2.08–3.17) *
Homemaker	0.34 (0.25–0.46) *	1.28 (1.16–1.41) *	1.04 (1.01–1.08) *	0.98 (0.95–1.01)	2.32 (2.13–2.53) *	2.56 (2.35–2.78) *	2.23 (1.84–2.71) *
Retired	1.62 (1.09–2.40) *	1.52 (1.28–1.80) *	1.02 (0.94–1.98)	1.11 (1.06–1.17) *	2.21 (1.95–2.51) *	2.13 (1.86–2.43) *	5.58 (4.44–7.02) *
Unemployed	1.33 (0.82–2.14)	1.22 (0.98–1.52)	1.03 (0.95–1.12)	1.10 (1.04–1.17) *	1.90 (1.62–2.22) *	2.08 (1.79–2.41) *	2.75 (1.98–3.82) *

*Significant Results: *P* value < 0.05; *PR* Prevalence Ratio

^a^Values in parentheses are 95% confidence intervals (CI)

^b^Raised blood pressure was defined as having blood pressure > 130/85 mmHg or taking an antihypertensive drug

^c^Participants with a body mass index ≥25 were classified as being overweight or obese

As age increased, there was also a significantly increased risk of consuming alcohol (PR: 1.33, 95% CI: 1.26–1.42), having a low intake of fruits and vegetables, and having a low level of physical activity, being overweight, and obese (PR: 1.44, 95% CI: 1.37–1.52), as well as presenting with abdominal obesity (PR: 1.50, 95% CI: 1.42–1.82) and high blood pressure (PR: 3.43, 95% CI: 3.05–3.85). Conversely, with increased age there was a decreased risk of smoking and alcohol consumption (Table 3).

People living in the tropics area had significantly higher risk of being a smoker (PR: 1.39, 95% CI: 1.13–1.70), being overweight and obese (PR: 1.32, 95% CI: 1.22–1.44), and presenting with abdominal obesity (PR: 1.49, 95% CI: 1.49–1.62), compared to those living in the Andean region (Table 3).

Compared to mestizos and whites, the indigenous participants had significantly higher risk of having a low intake of fruits and vegetables (PR: 1.08, 95% CI: 1.06–1.11) but lower risk of smoking (PR: 0.88, 95% CI: 0.79–0.98), alcohol consumption (PR: 0.94, 95% CI: 0.90–0.98), low levels of physical activity (PR: 0.89, 95% CI: 0.86–0.91), and having overweight and obesity (PR: 0.96, 95% CI: 0.92–0.99) (Table 3).

With lower education level, there also was a significantly decreased risk of being a smoker (PR: 0.47; 95% CI: 0.35–0.62) and of consuming alcohol (PR: 0.53; 95% CI: 0.47–0.59). However, people without formal schooling had a higher risk of having a low intake of fruits and vegetables (PR: 1.21; 95% CI: 1.15–1.26) and having raised blood pressure (PR: 1.24; 95% CI: 1.05–1.46) than those with higher education (Table 3).

Those who were currently married or in cohabitation had significantly higher risk of being an alcohol consumer (PR: 1.21; 95% CI: 1.15–1.27), being overweight and obese (PR: 1.67; 95% CI: 1.59–1.75), having abdominal obesity (PR: 1.75, 95% CI: 1.66–1.84), and having raised blood pressure (PR: 1.61, 95% CI: 1.31–1.41), than those who were never married, but their risk of low level of physical activity (PR: 0.89, 95% CI: 0.87–0.92) was significantly lower (Table 3).

All labour market position categories showed significantly higher risk of overweight and obesity, abdominal obesity, and raised blood pressure, compared to students. On the other hand, the risk of low level of physical activity was significantly lower in self-employees (PR: 0.62, 95% CI: 0.60–0.64) and employees (PR: 0.81, 95% CI: 0.77–0.85) when compared to students (Table 3).

## Discussion

This is the first STEPS survey conducted in Cochabamba and Bolivia. It provides current and accurate information about the prevalence of multiple cardiovascular risk factors and their social determinants. Our findings revealed that Cochabamba has a high prevalence of CVD risk factors, with a significant variation among the different socio-demographic groups. Indigenous populations and those living in the Andean region showed in general a lower prevalence for most of the risk factors evaluated.

Our findings pointed out a smoking prevalence lower than that estimated by the Bolivian Health Ministry and PAHO for Bolivia in 2015 (23.7%) but similar to the estimates for the Andean region (Colombia, Ecuador, Perú, Venezuela, and Bolivia) (12.2%) [6]. Smoking was higher among men (21.25%) than women (3.25%), which could be due to the social unacceptability of women’s use of tobacco in Bolivia and Latin America overall [6, 20–22]. However, exceptions have been found in Argentina (men: 29.5%, women: 18.4%), Chile (men: 40%, women: 36%), and Brazil (men: 19.3%, women: 11.3%), where women have high prevalence of smoking or the gender differences are smaller [6]. Our study also revealed a higher smoking prevalence related to increases in age and education, and among employed and single individuals, which is similar to the findings of the tobacco use survey in Central Asia and Latin America [23]. No comparable information exists about smoking in relation to ethnicity in LA; however, the low prevalence of smoking observed among indigenous populations (Quechua and Aymara) in our study could be explained by the common habit of chewing coca leaves [24, 25] among Andean indigenous communities. Traditionally, the coca leaves are said to have medicinal qualities and to provide energy [26], which is why they are used as a stimulant, especially among indigenous manual workers, including farmers and mine workers. Other factors explaining the low prevalence of smoking among indigenous people could be their low purchasing power and difficult access to cigarettes in rural areas [27]. As has been observed in Perú [28] and Brazil [29], the price of cigarettes and the population income modified the pattern of cigarette consumption, which could partially explain both the low prevalence of smoking among indigenous people, a group associated with low income, and the high prevalence of smoking among those with higher education level, usually a group with higher income [20, 29, 30].

The prevalence of alcohol consumption observed in Cochabamba was higher than those reported by other STEPS surveys worldwide [31–35]. However, the average amount of alcohol consumed in Bolivia (5.9 l/per person/year) is one of the lowest in South America and the Andean region (6.5 l/per person/year) [6]. Similar to our findings, other Latin-American studies have found a higher prevalence of alcohol use among men compared to women [36, 37], and in older age groups [38]. In Bolivia, previous studies have also found that alcohol consumption increases with age and has a high correlation with family abuse and poor school performance [39–41]. The lowest prevalence among indigenous people and those who live in the Andean region could be the result of disallowing alcohol sales and increasing the intolerance for drunken behaviour outdoors, as part of moral regulations introduced by evangelical movements in this population since the 1990s [42, 43].

The prevalence of low intake of fruit and vegetables in our study was high in all socio-demographic groups, except among those with higher education. Other surveys in Bolivia have found that diet relies heavily on potato, other tubers (54% of dietary energy), and grains (30% of dietary energy) [44], and suggest that Bolivian households of lower socioeconomic status prefer energy-dense and cheaper food sources [45]. These findings about dietary inadequacies could explain much of the higher prevalence of overweight and obesity found among participants in our study. Moreover, the low consumption of fruit and vegetables together with low levels of physical activity and a high burden of overweight and obesity in our population are a cause of concern, as they may lead to increased risk of CVD in the future. Low consumption of fruit and vegetables was particularly high among the indigenous population. This could be explained by the fact that members of this group consume mainly what they cultivate. Traditionally, in the Bolivian and Peruvian highlands, indigenous people plant potatoes, quinoa, and kañiwa (Andean legumes), as well as some barley, corn, and wheat [46]. Consequently, fruit and vegetables must be purchased from the lowlands or the tropics of Cochabamba, limiting the population’s access and the frequency of consumption recommended by WHO [44, 47].

Nearly two-thirds of the population in our study had a low level of physical activity, which was higher among women and older groups. The prevalence in our population was higher than the estimates from Ecuador (25.2%) but similar to the ones from Colombia (63.6%), according to a PAHO report [6]. Similar to our findings, that report pointed out that sedentary lifestyle is more prevalent among women than men, since most women are limited to working at home. These results are also similar to those reported in 2007 in a smaller population group in the capital of Cochabamba, where 62.2% of the participants were classified as sedentary [15]. Our findings on the prevalence of low physical activity point towards a growing increase of overweight and obesity, which should constitute a major concern for public health authorities.

Regarding overweight and obesity, the prevalence observed in Cochabamba was higher than in several other departments in the country—higher than the PAHO estimated average for Bolivia (men: 49%, women: 57.3%) but similar to the estimates for the Andean region (men: 55.1%, women: 60.0%) [6]. This high prevalence is probably related to the high consumption of carbohydrates and saturated fats in the usual diet profile of people living in Cochabamba [15]. In addition to the type of food, the common reuse of oils for frying and the increased trend of fast food consumption outside the home in this area [14] contribute to an increased caloric diet intake. However, in rural areas, especially in the Andean region, food is usually boiled instead of fried, which could explain the differences between the Andean region (inhabited mainly by indigenous communities) and the rest [44].

The prevalence of high blood pressure (17.15%) found in our study was low compared to previous studies conducted in Cochabamba (32%) [15] and other main cities in Bolivia, such as La Paz (34%) [48] and Santa Cruz (34.7%) [49]. However, these studies were carried out in hospital settings and mostly in urban areas, which could explain the differences with our findings [2, 5, 6]. On the other hand, similar to our findings, PAHO estimations in 2015 for Bolivia highlighted that high blood pressure was higher in men (19.7%) than in women (16.1%) [6].

## Limitations

The STEPS methodology is designed to provide standardized information on key modifiable risk factors that can be measured in population-based surveys without the need for high-technology instruments. Though the study provides reliable information, some limitations should be considered.

Even though the survey questions were adapted to the local context, some words or concepts may not have been understood in the same way by all participants, which could have introduced some potential bias. As the behavioural risk factors were self-reported, some of the information may have been concealed, especially information related to alcohol and tobacco use.

Although the anthropometric and blood pressure measurement instruments were periodically calibrated, the health personnel were adequately trained, and a survey implementation guide had been developed, the possibility for certain measurement errors cannot be discounted.

Despite these potential limitations, the large sample size and the inclusion of different subpopulations make our results generalizable to the Cochabamba and Bolivian context.

## Conclusion

Overall, our findings suggest a high prevalence of CVD risk factors in the population of Cochabamba, with significant variation among the different socio-demographic groups. Indigenous participants had a significantly lower risk of smoking, alcohol consumption, low levels of physical activity, overweight, and obesity, compared to mestizos and whites. Men, people living in the tropics area, and workers were found to have a significantly higher risk of smoking and alcohol use. The risks of having a low intake of fruit and vegetables increased significantly with age and decreased as education increased. The risk of sedentary lifestyle was significantly higher in people over 60 years of age or living in the tropics and Central Valley regions. Obesity and high blood pressure were significantly associated with age, residence, marital status, and occupation of the participants.

The information generated by this study provides evidence for health policy makers at the regional level and baseline data for department-wide action plans to carry out specific interventions at the population and individual levels. An increase in the burden of CVD could be expected if an effective multisectoral prevention strategy aimed at early diagnosis, monitoring, management, and prevention or control of cardiovascular risk factors is not implemented.

The results from this study also support the PAHO recommendations for strengthening the primary care systems in Bolivia and lay the groundwork for examining the financing, structure, and processes of care provided to patients with CVD in the region.

## Survey

In this study, we used the Spanish official version of the WHO-STEPS survey (V2.0). The content areas of the questionnaire as well as the practical guides to conduct the survey have been described in detail elsewhere [7].

## Data Availability

The datasets supporting the conclusions of this article are available upon request.

## Abbreviations

BHM: Bolivian health ministry
BMI: Body mass index
CVDs: Cardiovascular diseases
DALYs: Disability adjusted life years
GBD: Global burden of disease
GPAQ: Global physical activity questionnaire format
LA: Latin america
MET: Metabolic equivalent of task
NCD: Non-communicable diseases
NHIS: National health information system
PAHO: Pan American health organization
PHCSAs: Primary health care service areas
PSU: Population sampling unit
WHO: World Health Organization
